# Alterations in Gut Microbiota and Serum Metabolome Are Associated with Postpartum Depression

**DOI:** 10.3390/nu18152562

**Published:** 2026-08-05

**Authors:** Shengxuan Li, Min Pi, Zhuoxin Yang, Xiaoming Ma, Jinjun Yuan, Yumei Zhou

**Affiliations:** The Fourth Clinical Medical College of Guangzhou University of Chinese Medicine, Shenzhen 518033, China; 18980463263@163.com (S.L.); pppimin1992@163.com (M.P.); 001188@gzucm.edu.cn (Z.Y.); hhcaide@163.com (X.M.); yjjymx1302@163.com (J.Y.)

**Keywords:** postpartum depression, gut microbiota, serum metabolomics, gut–brain axis, short-chain fatty acids, lipid metabolism, nutritional metabolism

## Abstract

**Background:** Postpartum depression (PPD) is a prevalent and debilitating disorder, with increasing evidence implicating the gut microbiota–brain axis. However, integrated alterations in gut microbiota and circulating metabolites in PPD remain insufficiently characterized. **Methods:** Fecal and serum samples were collected from patients with PPD and healthy controls (HC). Depressive symptoms were assessed using the 17-item Hamilton Depression Rating Scale (HAMD-17). Gut microbiota was analyzed by 16S rRNA sequencing, and serum metabolites were profiled using untargeted LC–MS-based metabolomics. Spearman correlation and receiver operating characteristic (ROC) analyses were performed. **Results:** A total of 63 participants (42 PPD, 21 HC) were included. Significant alterations in gut microbial composition were observed in PPD, including decreased *Faecalibacterium* and *Akkermansia* and increased *Ralstonia* and *Fusobacterium*. Candidate differential serum metabolic features, including *LPE*-related and energy-metabolism-related features, were identified. Exploratory correlation analyses suggested distinct microbiota–metabolite association patterns, and several microbial taxa and serum metabolic features were associated with HAMD-17 scores. ROC analysis showed that several taxa and metabolic features exhibited preliminary discriminative performance with this cohort, although further validation is required. **Conclusions:** PPD was associated with alterations in gut microbiota composition and exploratory circulating metabolite profiles, potentially involving lipid dysregulation, neuroinflammation, steroid-related metabolism, and neurotoxicity-related pathways. The identified taxa and putatively annotated metabolites should be regarded as exploratory PPD-associated candidates rather than validated biomarkers or mechanistic mediators. Further validation in larger, independent cohorts is warranted. These findings may also inform future microbiota- and nutrition-oriented strategies for postpartum mental health management.

## 1. Introduction

Postpartum depression (PPD) is a common mental health condition after childbirth that can substantially impair maternal well-being and functioning, although effective treatments are available [1]. In low- and middle-income countries, prevalence estimates for PPD range from 6.5% to 12.9%, and may be considerably higher in certain settings [2,3,4]. For instance, a previous study reported an estimated prevalence of PPD in China was 21.4%, which was relatively high compared with estimates of 8.6% in the US and 14.0% in Japan [5]. In addition to sleep disturbances unrelated to infant care, symptoms of PPD frequently encompass anxiety, irritability, and a sense of overwhelm, along with preoccupation with the infant’s well-being and feeding [1]. Reports have also documented suicidal ideation and concerns regarding harm to the infant [6]. Consequently, PPD exerts profound adverse effects on patients’ physical and mental health.

The pathophysiology of PPD is complex and remains incompletely elucidated. Evidence indicates that biological factors, including hormonal impacts [7], genetics [8,9], and immune function [10], may substantially contribute. The gut–brain axis has therefore emerged as an important framework for investigating the biological processes underlying depression and for identifying new intervention strategies. Earlier research primarily emphasized endocrine communication between the digestive system and the central nervous system, whereas more recent studies have highlighted the contribution of gut microbial communities to the regulation of brain function and mental health [11,12]. The human gut microbiota, comprising trillions of microorganisms [13], may influence the central nervous system (CNS) through multiple indirect pathways, including immune, endocrine, metabolic, enteric nervous system, and vagal signaling [14,15]. Although peripheral serotonin (5-HT), GABA, dopamine, and acetylcholine can be produced or regulated in the gut, these gut-derived neuroactive compounds do not readily cross the blood–brain barrier (BBB) and should not be interpreted as directly acting within the brain [16,17,18,19,20,21,22]. Rather, they may contribute to gut–brain communication indirectly through local enteric, immune, endocrine, metabolic, and vagal pathways, whereas neurotransmitters functioning within the CNS are primarily synthesized locally in the brain. In addition, the gut microbiota can influence enteroendocrine cells through the production of microbial metabolites, such as secondary bile acids and short-chain fatty acids (SCFAs). SCFAs have received increasing attention as key microbial metabolites involved in depressive symptoms and gut–brain communication. Animal model studies have revealed a positive correlation between acetic acid concentrations and *Allobaculum* abundance [23]. Alterations in lipid metabolism have been associated with reduced levels of butyrate and acetate in experimental models of post-stroke depression [24]. Of note, the onset of depressive symptoms has been directly linked to decreased butyrate levels, a SCFA recognized for its anti-inflammatory properties [25]. Current evidence indicates that SCFAs are critical for maintaining blood–brain barrier (BBB) integrity [26,27], whereas BBB dysfunction has been implicated in the pathophysiology of depression [28,29]. Thus, metabolite profiles are intricately linked to gut microbial composition. Alterations in the microbial environment induce corresponding changes in metabolite profiles, thereby contributing to the pathogenesis of depression. Importantly, the gut microbiota and its metabolic output are highly responsive to dietary and nutritional factors [30,31]. During the postpartum period, women experience substantial changes in energy metabolism, lactation-related nutrient requirements, dietary patterns, and hormonal regulation, which may collectively reshape the gut microbiota and circulating metabolome [32,33]. Therefore, microbiota-derived metabolites, especially SCFAs, lipid-related metabolites, and steroid-related metabolites, may represent nutrition-sensitive biological links between postpartum metabolic status and depressive symptoms [34,35,36].

Our preliminary research [37] suggested that specific microbial taxa, including *Desulfovibrio* and *Paraprevotella*, may be involved in inflammation-related mechanisms and depressive symptoms through the gut–brain axis, and similar microbiota-related mechanisms have been reported previously [38]. Although microbiome and metabolome alterations have been increasingly reported in MDD and other depression-related conditions [39,40], integrative clinical evidence in PPD remains limited. Previous PPD studies have reported altered gut microbiota composition and associations with depressive symptoms and sex hormone levels [41]. Serum metabolomic studies have also suggested that PPD is accompanied by peripheral metabolic alterations. However, few clinical studies have simultaneously characterized gut microbiota and circulating metabolic features in the same postpartum cohort. Therefore, the present study aimed to extend existing PPD-related microbiome and metabolomics literature by integrating gut microbiota, serum metabolomics, and HAMD-17-based symptom assessment, providing exploratory evidence of postpartum-contextualized microbiome–metabolome patterns. Accordingly, comprehensive investigations integrating gut microbiota and metabolite profiling in postpartum clinical populations are warranted.

In this study, we integrated 16S rRNA gene sequencing with untargeted serum metabolomics using clinical samples from a Chinese cohort with PPD to characterize coordinated alterations in gut microbiota and circulating metabolites. We aimed to identify PPD-associated microbial taxa, differential serum metabolic features, and microbiota–metabolite association patterns, with particular attention to SCFA-producing taxa, lipid metabolism, steroid-related metabolism, inflammatory regulation, and nutrition-sensitive metabolic pathways.

## 2. Materials and Methods

### 2.1. Ethical Approval and Informed Consent

The research protocol received ethical approval from the Ethics Committee of Shenzhen Traditional Chinese Medicine Hospital [Shenzhen, China; Approval Code: K2020-027-01; Approval Date: 6 July 2020]. All procedures were designed and conducted in accordance with the Declaration of Helsinki. Eligible participants were fully informed of all procedures, potential benefits, and risks associated with the study and were permitted to withdraw at any time without providing a reason. Written informed consent was obtained before any study-related procedures were initiated.

### 2.2. Study Population and Sample Collection

This study included healthy controls and patients with PPD. All participants were recruited from Shenzhen Traditional Chinese Medicine Hospital and Shenzhen Maternity & Child Healthcare Hospital in Shenzhen, China. PPD was diagnosed according to the criteria outlined in the Fourth Edition of the Diagnostic and Statistical Manual of Mental Disorders (DSM-5). Participants who had delivered within the preceding year were eligible for further assessment. Recruitment for this study was conducted between January 2021 and December 2021.

The criteria for patients with PPD were as follows: (1) aged 20 to 49 years; (2) diagnosis of PPD confirmed by a psychiatrist; (3) onset of illness occurring within 12 months postpartum; (4) a score of 7 to 24 on the 17-item Hamilton Depression Rating Scale (HAMD-17); and (5) provision of written informed consent.

Patients meeting any of the following criteria were excluded from the study: (1) diagnosis of bipolar disorder (according to DSM-5 criteria) or other severe mental disorders, such as schizophrenia; (2) cognitive impairment, characterized by difficulty comprehending questionnaire materials due to neurological disorders or other factors, or inability to participate in a productive interview; (3) current pregnancy; (4) a score greater than 2 on the suicide item of the HAMD-17; (5) a history of suicide attempt within the past year; or (6) a diagnosis of anorexia nervosa.

The inclusion criteria for healthy volunteers were as follows: (1) age between 20 and 49 years; (2) absence of any discernible physical or psychological discomfort; (3) standard postpartum health assessment within normal limits, including routine blood count, liver and kidney function tests, and electrocardiogram; (4) a score of less than 7 on the HAMD-17; and (5) provision of written informed consent for voluntary participation in the study.

Individuals meeting any of the following criteria were excluded from the study: (1) current pregnancy; (2) a history of suicide attempt within the preceding year; (3) a score greater than 2 on the suicide item of the HAMD-17; or (4) concurrent participation in other clinical trials.

### 2.3. Evaluation of Clinical Scales

All participants completed the assessment procedures using the HAMD-17 and the Structured Clinical Interview for the Fourth Edition of the DSM-5. The HAMD-17 is a 17-item clinician-rated instrument designed to assess the severity and frequency of depressive symptoms in individuals with major depressive disorder (MDD) [42]. The HAMD-17 scores vary from 0 to 52: “7 < scores ≤ 17” indicates clinically mild depression; “17 < scores ≤ 24” is indicative of moderate depression; “scores > 24” denotes severe depression.

### 2.4. Statistical Analysis of Clinical Characteristics

Between-group comparisons of demographic and clinical variables were conducted using SPSS 26.0 software (IBM, Armonk, NY, USA). The distribution of each continuous variable was assessed before statistical testing. Normally distributed variables were summarized as mean ± standard deviation and compared using an independent-samples *t*-test. Variables with non-normal distributions were expressed as median and interquartile range (IQR) and analyzed using the Mann–Whitney U test. Categorical variables were analyzed using the chi-square test or Fisher’s exact test, as appropriate, and are presented as frequencies and percentages. All tests were two-sided, with *p* < 0.05 indicating statistical significance.

### 2.5. Sample Collection and Processing

Fecal samples were collected from all participants within two days following the HAMD-17 assessments. Samples were placed in sterile plastic containers immediately after defecation and temporarily stored at −20 °C for no longer than half a day. All fecal samples were subsequently transported at −80 °C to the laboratories of Shenzhen Traditional Chinese Medicine Hospital and Shenzhen Maternity & Child Healthcare Hospital for hospitalized patients within the same day. Upon arrival, samples were preserved in a −80 °C freezer. Detailed procedures for fecal sample collection have been described previously [43]. Blood samples were centrifuged to obtain serum, and 0.5 mL aliquots were transferred into 1.5 mL centrifuge tubes. Prior to use, serum sample tubes were labeled and stored at −80 °C.

### 2.6. DNA Extraction

DNA extraction was performed using the MO BIO PowerSoil^®^ DNA Isolation Kit (MO BIO Laboratories, Inc., Carlsbad, CA, USA), and the extracted DNA was stored at −80 °C in Tris-EDTA buffer solution (Sigma-Aldrich, St. Louis, MO, USA). To amplify the V4 region of the 16S rRNA gene and incorporate barcode sequences, custom fusion primers were designed based on the universal primer set 515F (5′-GTGYCAGCMGCCGCGGTAA-3′) and 806R (5′-GGACTACNVGGGTWTCTAAT-3′), combined with barcode sequences. PCR mixtures were prepared with a final volume of 50 μL, comprising 1 μL of each forward and reverse primer (10 μM), 1 μL of template DNA, 4 μL of dNTPs (2.5 mM each), 5 μL of 10× EasyPfu Buffer (TransGen Biotech Co., Ltd., Beijing, China), 1 μL of EasyPfu DNA Polymerase (2.5 U/μL), and 1 μL of double-distilled water. Thermal cycling conditions were as follows: initial denaturation at 95 °C for 5 min; followed by 30 cycles of denaturation at 94 °C for 30 s, annealing at 60 °C for 30 s, and extension at 72 °C for 40 s; and a final extension step at 72 °C for 4 min. Amplicons were analyzed by agarose gel electrophoresis. The expected amplicon size for the 515F-806R primer pair was approximately 300–350 bp. Amplicons were quantified using the Quant-iT™ PicoGreen^®^ dsDNA Assay Kit (P11496; Thermo Fisher Scientific, Waltham, MA, USA) in accordance with the manufacturer’s instructions. For high-throughput sequencing on the Illumina MiSeq platform (Illumina, Inc., San Diego, CA, USA) using V3 reagent chemistry (PE150, 300 cycles), amplicon libraries were pooled in equimolar amounts and subsequently quantified using the KAPA Library Quantification Kit (KK4824; Illumina, Inc., San Diego, CA, USA) in accordance with the manufacturer’s protocols.

### 2.7. Metabolomics Preparation for Serum Samples

A 50 μL aliquot of each sample was combined with 200 μL of extraction solution (acetonitrile: methanol = 1:1, *v*/*v*) containing isotopically labeled internal standards. The mixture was vortex-mixed for 30 s, sonicated in an ice-water bath for 10 min, and incubated at −40 °C for 1 h to precipitate proteins. The mixture was centrifuged at 12,000 rpm (13,800× *g*) for 15 min at 4 °C, and the supernatant was subsequently collected for LC–MS-based analysis. A pooled quality control (QC) sample was generated by combining equal aliquots of supernatant from all individual samples. Chromatographic separation was performed using a Vanquish UHPLC system (Thermo Fisher Scientific) equipped with a Waters ACQUITY UPLC BEH Amide column. The mobile phase consisted of water (A) and acetonitrile (B), and the injection volume was 2 μL at a column temperature of 4 °C. Mass spectrometry analysis was performed using an Orbitrap Exploris 120 mass spectrometer (Thermo Fisher Scientific, Bremen, Germany) controlled by Xcalibur software (version 4.4). MS^1^ and MS^2^ data were acquired under the following parameters: sheath gas flow rate of 50 arbitrary units (Arb), auxiliary gas flow rate of 15 Arb, capillary temperature of 320 °C, and spray voltages of 3.8 kV in positive ion mode and −3.4 kV in negative ion mode. Raw data were converted to mzXML format using ProteoWizard (version 3.0.21229) and subsequently processed through a custom XCMS-based R pipeline for automated peak picking, alignment, and integration. Compound identification was performed by matching against an in-house MS^2^ spectral database.

### 2.8. Statistical Analyses

#### 2.8.1. Clinical Parameters

Statistical analysis of clinical parameters was performed using SPSS 26.0 software. Normality tests were initially conducted on continuous variables. Normally distributed continuous data are presented as mean ± standard deviation (SD). The Wilcoxon rank-sum test was used to compare variables with unequal variances, whereas the *t*-test was applied to data meeting the assumption of homogeneity of variance. For non-normally distributed variables, group comparisons were performed using the Wilcoxon rank-sum test, and results are presented as median values. Categorical variables were analyzed using the chi-square test or Fisher’s exact test, as appropriate, and are presented as frequencies and percentages. A statistically significant difference was defined as a *p* Value less than 0.05.

#### 2.8.2. Sequencing Data Analysis

Raw paired-end sequences were demultiplexed according to barcode information and subjected to quality filtering. Low-quality reads, ambiguous sequences, and reads with mismatched barcodes or primers were removed. Paired-end reads were merged based on overlapping regions. Operational taxonomic units (OTUs) were clustered at a 97% sequence similarity threshold using UPARSE. Chimeric sequences were identified and removed during the OTU clustering process. Representative OTU sequences were taxonomically assigned against the SILVA database. Richness, Chao1, and Shannon_2 were used to estimate the α-diversity. β-diversity was assessed using the Canberra distance metric, and principal coordinate analysis (PCoA) was applied to visualize the resulting distance matrices. Group differences in β-diversity were tested using permutational multivariate analysis of variance (PERMANOVA; Adonis test) implemented in the vegan package in R, with R^2^ used as the effect size. To further identify candidate discriminative bacterial taxa at the genus level, linear discriminant analysis effect size (LEfSe) was applied through the Huttenhower Lab Galaxy Server [44] after taxa summaries were reformatted. In the setting of LEfSe, firstly, the Kruskal–Wallis’s test (α = 0.05) was employed to detect taxa using differential abundance analysis; secondly, the Wilcoxon rank-sum test was used to investigate the biological consistency among subclasses. Finally, the effect size of differentially abundant genera was estimated by linear discriminant analysis (LDA) [44], and the threshold on the logarithmic LDA score for discriminative features was 2.5. All the analyses were conducted using the “vegan” package in R 3.4.1 software.

#### 2.8.3. Untargeted Metabolomics Data Analysis

All metabolite annotations were validated by cross-referencing them with entries in the Human Metabolome Database (http://www.hmdb.ca, accessed on 20 January 2022) and METLIN (http://metlin.scripps.edu, accessed on 20 January 2022). The datasets were examined utilizing MetaboAnalyst R [45]. Prior to OPLS-DA, the data were log2-transformed and mean-centered. OPLS-DA models were used to generate score plots and calculate variable importance in VIP values. Model robustness and the potential for overfitting were assessed using a permutation test with 200 permutations. Candidate differential serum metabolic features were screened using the combined criteria of VIP ≥ 1, an absolute log2 fold change (|log2FC|) ≥ 0.585, and a nominal *p* Value < 0.05 obtained using Student’s *t*-test. The *p* Values used for candidate feature screening were unadjusted, and false discovery rate correction was not applied. Accordingly, features meeting these criteria were interpreted as exploratory candidates rather than statistically confirmed differential metabolites. Box plots were generated to visualize the distributions of selected metabolic features. Data processing and visualization were performed using the reshape2 and ggpubr packages in R.

#### 2.8.4. Clinical Variables and Integrative Analyses

**Figure 1:**
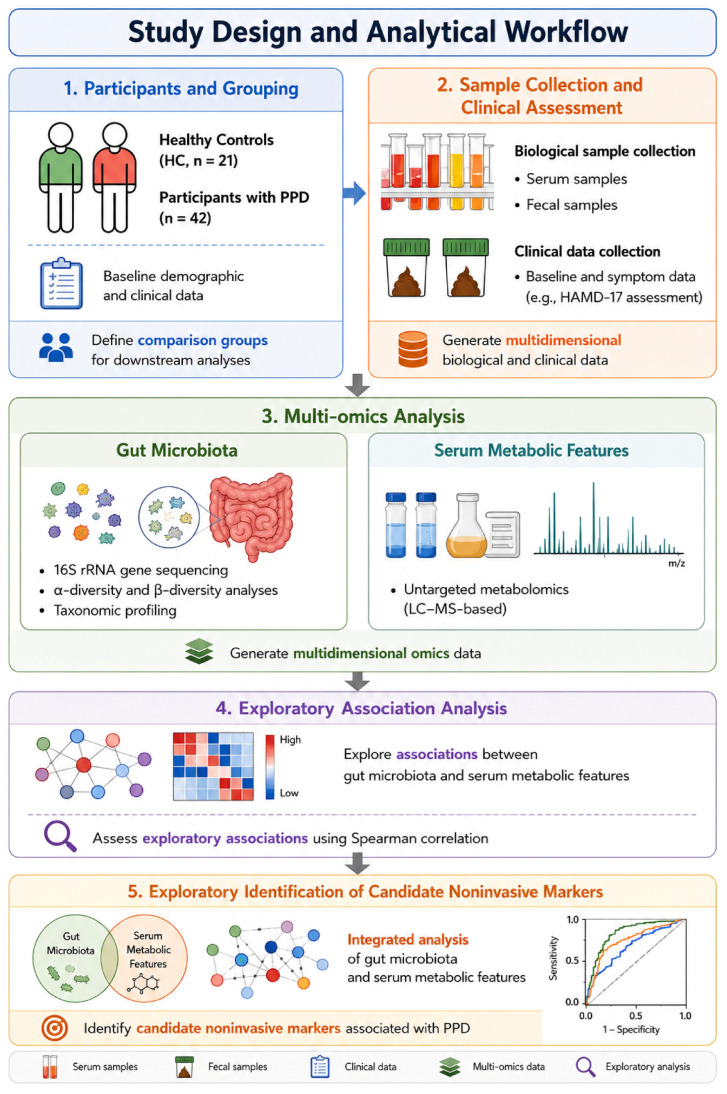
Schematic overview of the study design, sample collection, multi-omics analysis, and biomarker identification workflow.

Subsequent analyses were performed using R software. Categorical variables are presented as frequencies (percentages). For continuous variables, data with non-normal distributions are presented as medians with IQR. To compare differences among study groups, the chi-square test was used for categorical variables, and the Mann–Whitney U test was applied for non-normally distributed continuous variables. Correlations between gut microbiota, metabolites and HAMD-17 scores were evaluated using Spearman’s rank correlation coefficient. The *p* Values obtained from these correlation analyses were nominal and unadjusted, and false discovery rate correction was not applied. Accordingly, the identified associations were interpreted as exploratory and hypothesis-generating. To visualize the results, heatmaps and Sankey diagrams were generated. Finally, candidate discriminative features, including selected bacterial taxa and serum metabolic features, were further evaluated using receiver operating characteristic (ROC) curve analysis, with the area under the curve (AUC) used to assess exploratory diagnostic value. To assess the internal robustness of the ROC results and reduce the risk of overfitting, bootstrap resampling with 1000 iterations was performed, and bootstrap-derived 95% confidence intervals were calculated for the AUC estimates. In each bootstrap iteration, samples were resampled with replacement, and the AUC was recalculated. Given the exploratory nature of this study and the relatively limited sample size, ROC analyses were performed to assess the preliminary discriminative performance of selected microbial taxa and serum metabolites. These analyses were not intended to establish validated diagnostic models, and the identified candidate biomarkers require further validation in independent cohorts. The procedural steps and methodologies of our study are elucidated in a flowchart depicted in Figure 1, providing a clear visual representation of the study’s analytical framework.

## 3. Results

### 3.1. Demographic and Clinical Characteristics of the Participants

Between January 2021 and December 2021, a total of 63 women were enrolled at Shenzhen Traditional Chinese Medicine Hospital and Shenzhen Maternity & Child Healthcare Hospital. The final study cohort comprised 42 participants with PPD and 21 HCs. All participants completed the HAMD-17 evaluations and provided serum and fecal samples for subsequent analyses.

Demographic variables, including age, body mass index (BMI), postpartum duration, educational background, menstruation status, constipation, multiparity, breastfeeding status, cesarean delivery, and irregular dietary pattern did not differ significantly between the HC and PPD groups (Table 1; *p* > 0.05). The overall scores for HAMD-17 were considerably elevated in the PPD group compared to the HC group (Table 1; *p* < 0.01). Additional baseline information was collected, including specialized diets, gastrointestinal disorders, metabolic abnormalities, and prior use of antibiotics or probiotics. Based on questionnaire responses, no participants reported a history of irritable bowel syndrome or colon cancer; however, constipation was reported by a subset of individuals (Table 1). Furthermore, all participants were long-term residents of Shenzhen, China. Based on self-reported habitual eating patterns, they generally consumed relatively light and easily digestible meals, with limited intake of greasy, energy-dense, and spicy foods. Most participants also reported a broadly balanced diet that included both vegetables and animal-derived foods. Additionally, all participants reported no recent consumption of antibiotics, probiotics, or traditional Chinese medicines and were free from gastrointestinal disorders.

### 3.2. Collection of 16S Ribosomal RNA (rRNA) Sequences

In this study, a total of 63 participants provided fecal samples that were successfully analyzed by 16S rRNA gene sequencing. After quality filtering, 5,538,367 high-quality sequences were retained from 5,553,309 raw sequences. Subsequently, a total of 3339 OTUs were clustered for downstream analyses. Sequencing data were obtained from 42 patients with PPD and 21 healthy controls.

### 3.3. Analysis of Microbial α- and β-Diversity

**Figure 2:**
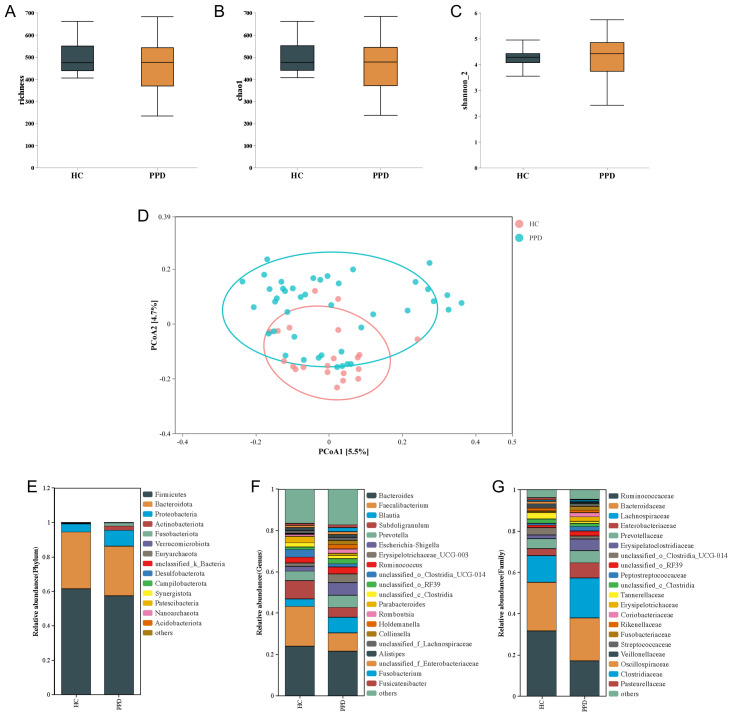
Gut microbiota diversity and taxonomic composition in PPD and HC. (**A**–**C**) Microbial α-diversity, including (**A**) richness index, (**B**) Chao1 index, and (**C**) Shannon diversity. (**D**) Microbial β-diversity based on the Canberra distance metric. Group differences were assessed using the Wilcoxon rank-sum test. (**E**–**G**) Taxonomic composition of gut microbiota at different levels: (**E**) phylum, (**F**) genus, and (**G**) family.

Gut microbial α-diversity and β-diversity profiles are summarized in Figure 2. No statistically significant between-group differences were detected in observed richness, Chao1, or Shannon_2 indices (*p* = 0.058, 0.059, and 0.684, respectively) (Figure 2A–C). Nonetheless, the richness and Chao1 indices appeared to be higher in the HC group than in the PPD group (Figure 2A,B). PCoA was performed to evaluate differences in β-diversity between the two groups using the Canberra distance metric (Figure 2D). As shown in Figure 2D, PERMANOVA based on the Canberra distance matrix showed a significant difference in bacterial community composition between the PPD and HC groups (Adonis: *F* = 1.950, *R^2^* = 0.031, *p* = 0.001). The first two PCoA axes explained 5.51% and 4.67% of the total variation, respectively. The findings indicated that fecal bacterial β-diversity indices were more tightly clustered in the HC group compared to the PPD group.

### 3.4. Composition of Microbial Communities

Taxonomic profiles were visualized using stacked bar plots of relative abundance at the phylum, family, and genus levels (Figure 2E–G). These plots summarized the overall distribution of the predominant microbial taxa in the PPD and HC groups. Analysis of gut microbiota composition at the phylum and genus levels provided an overview of the overall microbial structure. Notable differences in microbial composition were observed at the genus level (Figure 2F). *Bacteroides* was the most dominant genus, accounting for 24.01% of sequences in the HC group and 21.57% in the PPD group. At the family level, *Ruminococcaceae* exhibited a higher relative abundance in the HC group compared to the PPD group (Figure 2G). At the phylum level, *Nanoarchaeota* and *Acidobacteriota* were detected exclusively in the PPD group, although their relative abundances were low (Figure 2E).

### 3.5. Comparison of Bacterial Genera Between HC and PPD

**Figure 3:**
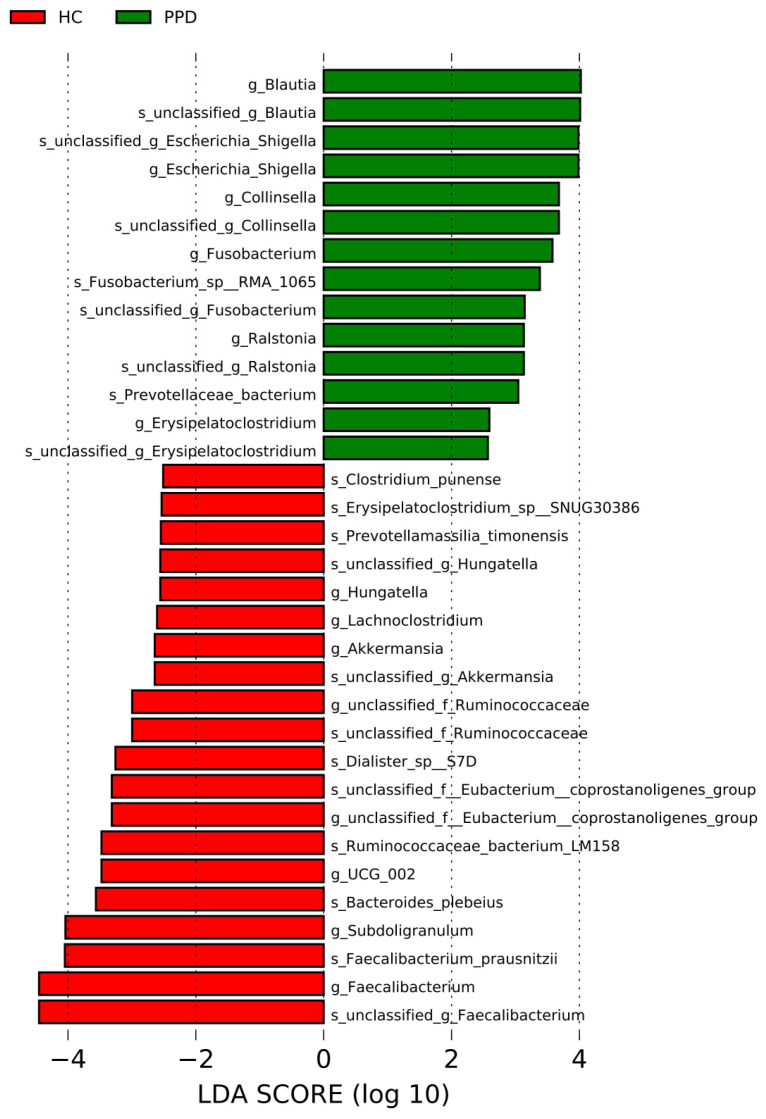
Differential gut microbial taxa between HC and PPD groups identified by LEfSe analysis Only genera with a LDA score > 2.5 are shown.

**Figure 4:**
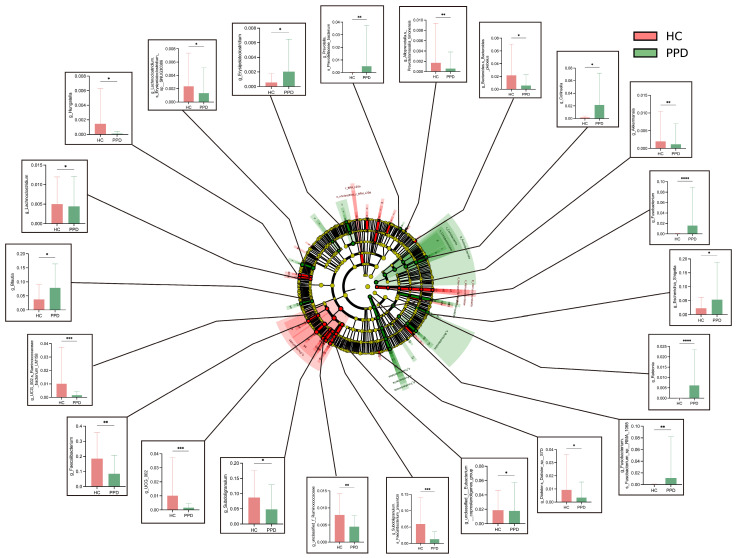
Differentially abundant bacterial taxa between HC and PPD groups identified by LEfSe analysis. Cladogram illustrating taxa with the greatest differences in relative abundance. Red indicates taxa enriched in the HC group, whereas green indicates taxa enriched in the PPD group. * *p* < 0.05, ** *p* < 0.01, *** *p* < 0.001, and **** *p* < 0.0001.

To delineate taxa contributing to the observed between-group differences, LEfSe analysis was conducted to identify candidate discriminative bacterial features associated with PPD using a nominal *p* value < 0.05 and an LDA score > 2.5. In the presented results, the color scheme corresponds to group-specific enrichment: green corresponds to microbiota enriched in the PPD cohort, and red to those enriched in the healthy cohort. *Faecalibacterium*, *Faecalibacterium_prausnitizii*, *Subdoligranulum*, *Bacteroides_plebeius*, *UCG_002*, *Ruminococcaceae_bacterium_LM158*, *Akkermansia*, *Lachnoclostridium*, *Hungatella*, *Prevotellamassilia_timonensis*, and *Clostridium_punense* were mainly enriched in the HC group. *Blautia, Escherichia_Shigella*, *Collinsella*, *Fusobacterium*, *Ralstonia*, *Prevotellaceae_bacterium*, and *Erysipelatoclostridium* were mainly enriched in the PPD group (Figure 3 and Figure 4).

### 3.6. Exploratory Serum Metabolic Features Associated with PPD

**Figure 5:**
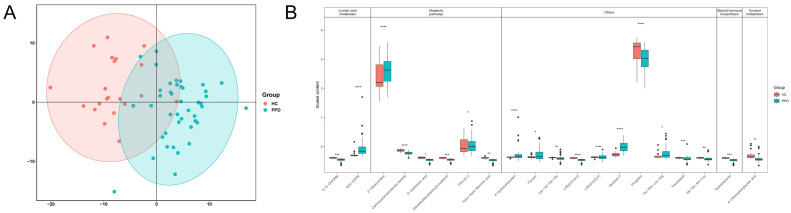
Serum metabolomic alterations in PPD and HC. (**A**) OPLS-DA score plot of serum metabolites showing separation between PPD and HC. (**B**) Differential serum metabolites between the PPD and HC. * *p* < 0.05, ** *p* < 0.01, *** *p* < 0.001, and **** *p* < 0.0001.

To investigate alterations in serum metabolites associated with PPD, serum metabolite profiling was performed using untargeted LC–MS-based metabolomics. OPLS-DA score plots based on serum metabolites clearly discriminated between the HC and PPD groups, indicating distinct metabolic profiles. Pathway analysis suggested that the candidate differential serum metabolic features were primarily related to four pathways, including linoleic acid metabolism, steroid hormone biosynthesis, tyrosine metabolism, and general metabolic pathways. Compared with HC, levels of *9(S)-HODE*, *2’-Deoxyuridine*, *4-Chlorophenylacetic acid*, *Flunixin*, *LPE(0:0/22:4), and Penitrem F were significantly increased in the PPD group, whereas 9,10-DiHOME*, *Carboxyaminoimidazole ribotide*, *D-Arabinonic acid*, *Demethyldecarbamoylnovobiocin*, *FFA (16:1)*, *LPE(0:0/16:0)*, *Progoitrin*, and *Testololactone* were significantly decreased (Figure 5). These results were based on nominal, unadjusted *p* Values and should be interpreted as exploratory.

### 3.7. Exploratory Associations Among Gut Microbiota, Serum Metabolic Features, and HAMD-17 Scores

**Figure 6:**
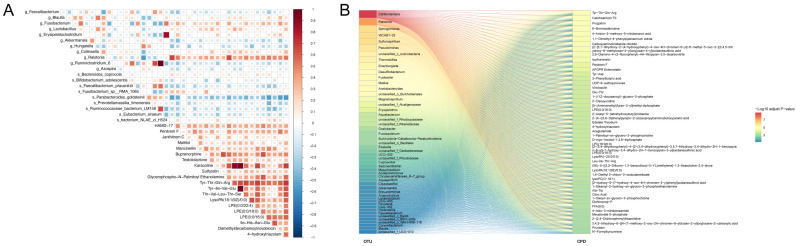
Exploratory associations among gut microbiota, serum metabolites, and HAMD-17 scores in PPD. (**A**) Heatmap showing Spearman correlation coefficients among differential microbial taxa, serum metabolites, and HAMD-17 scores. (**B**) Sankey diagram illustrating significant microbiota–metabolite associations identified based on 150 bootstrap iterations.

Spearman’s correlation analysis was used to explore relationships among differential gut microbial taxa, serum metabolic features, and HAMD-17 scores. At the genus level, *Ralstonia* showed nominal positive associations with several serum metabolic features, with the strongest nominal association observed for *Tyr-Thr-Gln-Arg*. Additional nominal positive associations were observed between selected serum metabolic features and several bacterial genera, including *Fusobacterium*, *Lactobacillus*, *Erysipelatoclostridium*, and *Collinsella*. However, *Parabacteroides_goldsteinii*, *Prevotellamassilia_timonensis*, *Ruminococcaceae* and *Eubacterium_siraeum* all showed nominal inverse associations with serum metabolites. In addition, HAMD-17 scores showed positive associations with taxa, particularly *Ralstonia*, and *Fusobacterium*. In contrast, negative associations were observed between HAMD-17 scores and several taxa enriched in the HC group, including *Hungatella*, *Faecalibacterium*, and *Akkermansia*. HAMD-17 scores were also positively associated with several serum metabolic features, including *LPE-related metabolites*, *Tyr-Thr-Gln-Arg*, *Penitrem F, and Testololactone*. These associations were identified using unadjusted nominal *p* Values and should therefore be interpreted as exploratory and hypothesis-generating. Figure 6 displays the heatmaps and Sankey diagrams generated from the correlation analysis.

### 3.8. Exploratory Evaluation of Discriminative Microbial and Serum Metabolic Features Associated with PPD

Based on LEfSe and differential metabolite analyses, ROC analyses were performed to evaluate the exploratory discriminative performance of selected microbial taxa and serum metabolic features associated with PPD. Microbiome and metabolite data were standardized, and features were grouped into three categories. Features with an AUC > 0.70 were selected for exploratory evaluation. ROC curves were generated using the scikit-learn package. To assess the internal robustness of the ROC-based results and reduce the risk of overfitting, bootstrap resampling with 1000 iterations was performed.

**Figure 7:**
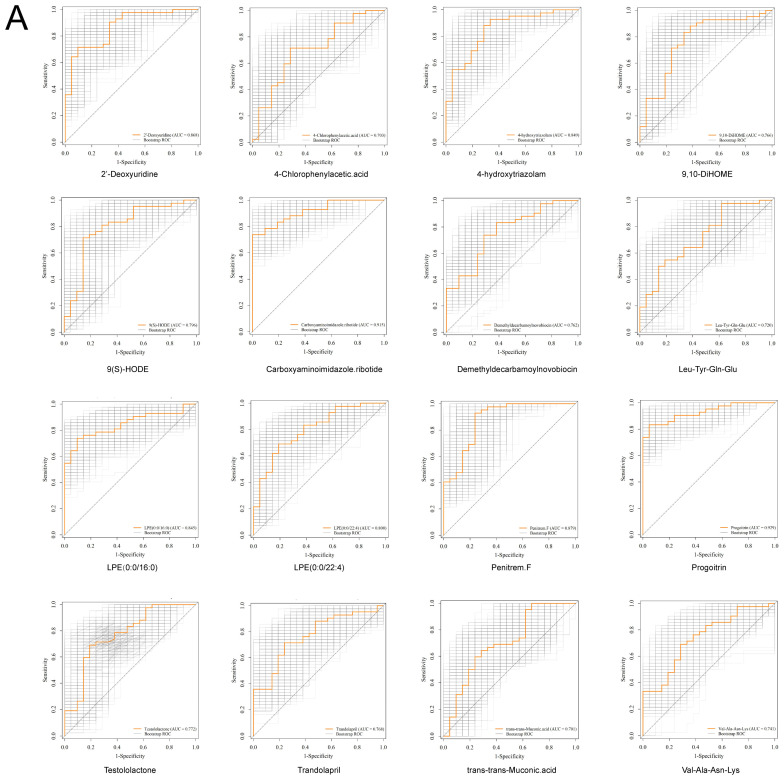

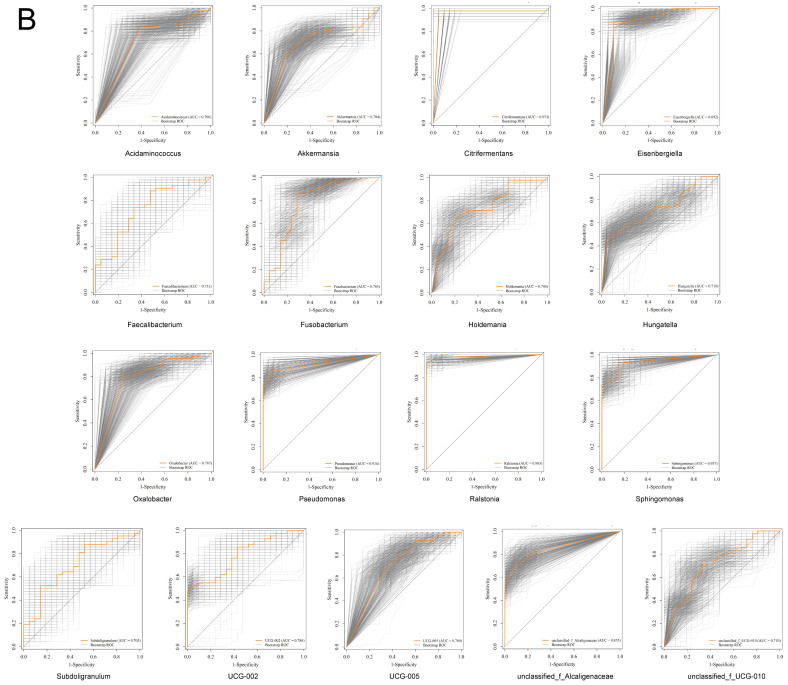
Exploratory ROC analysis of selected serum metabolic features and gut microbial taxa associated with PPD. (**A**) ROC curves of selected serum metabolic features showing their preliminary discriminative performance. (**B**) ROC curves of selected gut microbial taxa. Only features with an area under the curve (AUC) > 0.70 are shown. Bootstrap resampling with 1000 iterations was performed to assess the internal stability of the ROC results. The corresponding bootstrap-derived 95% confidence intervals for the AUC estimates are provided in Supplementary Table S1. The dashed line represents random classification. These findings reflect preliminary within-cohort discriminative performance and should not be interpreted as externally validated diagnostic models.

Several metabolic features exhibited exploratory discriminative performance after bootstrap internal validation, including *Progoitrin* (AUC = 0.929), *Carboxyaminoimidazole ribotide* (AUC = 0.915), *Penitrem F* (AUC = 0.879), *4-hydroxytriazolam* (AUC = 0.849), *LPE (0:0/16:0)* (AUC = 0.845), *2’-Deoxyuridine* (AUC = 0.868), *LPE (0:0/22:4)* (AUC = 0.8), *9(S)-HODE* (AUC = 0.796), *Testololactone* (AUC = 0.772), *Trandolapril* (AUC = 0.768), *Demethyldecarbamoylnovobiocin* (AUC = 0.762), *9,10-DiHOME* (AUC = 0.766), *Val-Ala-Asn-Lys* (AUC = 0.741), *Leu-Tyr-Gln-Glu* (AUC = 0.72), *4-Chlorophenylacetic acid* (AUC = 0.703), and *trans-trans-Muconic acid* (AUC = 0.701) (Figure 7A).

Similarly, multiple microbial taxa showed exploratory discriminative performance, particularly *Ralstonia* (AUC = 0.983), *Citrifermentans* (AUC = 0.953), *Sphingomonas* (AUC = 0.937), *Pseudomonas* (AUC = 0.916), and *Eisenbergiella* (AUC = 0.892). Other taxa, including *unclassified_f_Alcaligenaceae* (AUC = 0.885), *UCG-002* (AUC = 0.788), *Oxalobacter* (AUC = 0.787), *Fusobacterium* (AUC = 0.785), *Holdemania* (AUC = 0.74), *Hungatella* (AUC = 0.718), *Faecalibacterium* (AUC = 0.713), *unclassified_f_UCG-010* (AUC = 0.71), *UCG-005* (AUC = 0.709), and *Acidaminococcus* (AUC = 0.706), *Subdoligranulum* (AUC = 0.705), and *Akkermansia* (AUC = 0.704), also demonstrated acceptable exploratory discriminative performance (Figure 7B).

## 4. Discussion

**Figure 8:**
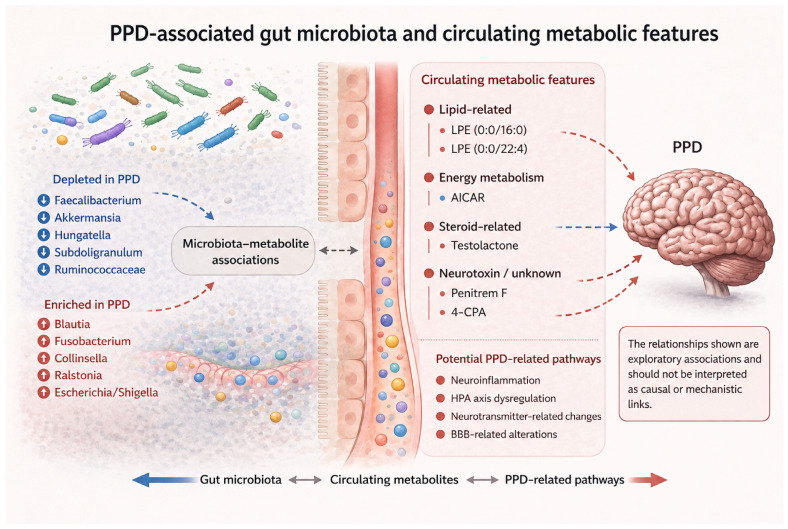
Conceptual summary of potential associations among altered gut microbiota, serum metabolic features, and PPD-related biological processes. *Penitrem F* and *Testololactone* represent putative and analytically unconfirmed annotations from untargeted LC–MS analysis; their inclusion in this conceptual figure does not establish their chemical identities, biological origins, or mechanistic roles in PPD. The pathways shown are literature-informed hypotheses provided for biological context; they were not directly tested in this study and should not be interpreted as causal or mechanistic relationships.

Accumulating evidence indicates that disturbances in the intestinal microbial community are associated with PPD [46]., suggesting that altered host–microbe communication may contribute to its biological basis. Circulating metabolites related to host–microbial co-metabolism may provide a possible biological context for microbiota–brain communication. However, the present study did not determine the microbial origin of the detected serum metabolic features or demonstrate their regulatory effects in PPD [36]. In the present study, fecal microbial profiles were compared between participants with PPD and healthy controls using 16S rRNA gene sequencing, while serum metabolic alterations were examined through untargeted metabolomics. We subsequently explored associations between differential microbial taxa and circulating metabolic features. Finally, ROC curve analyses were also performed to evaluate the predictive performance of these microbial and metabolic features. Our findings suggest that combined assessment of gut microbiota and serum metabolites may serve as a noninvasive exploratory approach for PPD (Figure 8).

The principal contribution of this study lies not in the use of microbiome or metabolomics analysis alone, as integrated microbiome–metabolome approaches have already been applied in MDD [39,40]. Rather, this study jointly characterized gut microbial composition, circulating serum metabolic features, and HAMD-17-based symptom severity within the same postpartum cohort. In contrast, available PPD studies have more commonly examined maternal gut microbiota or serum metabolomic alterations separately [37,47]. This integrated design provides exploratory cross-domain association patterns within a postpartum context shaped by nutritional, lactation-related, and microbiome-associated metabolic conditions [32,33,34,35,36]. However, because a non-postpartum MDD comparison group was not included, these findings should be regarded as postpartum-contextualized rather than PPD-specific.

### 4.1. Gut Microbiota Analysis

Gut microbiota diversity, encompassing both the richness and evenness of microbial species distribution, is typically partitioned into α-diversity and β-diversity and has increasingly been recognized as a key biomarker in the expanding literature on depression [48,49,50]. α-diversity is widely employed as an index of microbial community stability and functional capacity, both of which are thought to confer benefits to the host [51].

No clear consensus has been reached regarding differences in α-diversity. One study reported that, among patients with MDD, non-responders to antidepressant treatment exhibited higher intestinal microbial α-diversity than responders, relative to HC [52]. By contrast, recent findings indicate Shannon and Simpson indices do not differ significantly between individuals with MDD and those without depression [53]. Consistent with the latter findings, our study did not detect a significant difference in α-diversity between the PPD group and HC. Our previous findings are consistent with this pattern, demonstrating no significant differences in α-diversity [37]. β-diversity characterizes inter-sample variation in microbial community composition and relative abundance [54]. In the present study, β-diversity revealed a significant difference in overall gut microbial community structure between participants with PPD and HC. Comparable alterations in between-group microbial composition has also been documented in individuals with depression by Kelly et al. and Zheng et al. [55,56].Although age, BMI, postpartum duration, mode of delivery, and broadly assessed dietary characteristics did not differ significantly between groups, the observed β-diversity difference may reflect PPD-associated microbial community reorganization, potentially related to inflammatory, neuroendocrine, and metabolic alterations rather than measured demographic or broad dietary factors [46,57,58]. Gut microbiota diversity is influenced by multiple factors, including age, diet, and overall health status. A substantial body of evidence suggests that greater microbial diversity is associated with protection against autoimmune and metabolic disorders [59,60,61]. However, some studies have reported reduced microbial diversity in individuals with MDD [62]. Additionally, significant differences in gut microbiota diversity have been observed between individuals with bipolar disorder and HC [63].

Our findings demonstrated significant differences in both the richness and composition of gut microbial communities between the PPD and HC group. Specifically, *Blautia*, *Escherichia_shigella*, *Collinsella*, *Fusobacterium*, *Ralstonia*, *prevotellaceae*, and *Erysipelatoclostridium* were enriched in the PPD group. These taxa have been previously associated with a range of neuropsychiatric and gastrointestinal disorders, including depression [64,65,66,67,68], MDD, autism spectrum disorder (ASD) [69], MDD [70], and irritable bowel syndrome (IBS) [71]. Notably, *Escherichia_shigella*, a member of the Enterobacteriaceae family and a common constituent of the gut microbiota, may promote intestinal inflammation and increased gut permeability when overabundant, thereby facilitating microbial translocation and systemic inflammation [72]. Likewise, Fusobacterium, a Gram-negative anaerobe, exhibits invasive and pro-inflammatory properties linked to immune activation [73,74]. This interpretation is supported by evidence linking depression to a state of chronic, mild systemic inflammation, as reflected by elevated circulating IL-1β, IL-6, and other pro-inflammatory mediators [75,76].

Supporting this, increased Fusobacterium abundance has been reported in anxiety and ASD cohorts [64,69]. In addition, elevated levels of *Blautia* [65,66,67], *Collinsella* [68,77], and *Ralstonia* [70] have been documented in individuals with anxiety and depressive disorders, in agreement with our findings. Together, these results suggest that alterations in gut microbial composition may contribute to PPD through inflammation-related mechanisms within the gut–brain axis.

Several taxa were enriched in the HC group, including *Faecalibacterium, Subdoligranulum, Bacteroides_plebeius, UCG_002, Ruminococcaceae, Akkermansia, Lachnoclostridium,* and *Hungatella*, consistent with previous observational studies. In other words, these taxa were observed to be reduced in the PPD group. Notably, several of these genera are recognized as producers of short-chain fatty acids (SCFAs), particularly butyrate, and may contribute to the preservation of intestinal barrier integrity and immune balance. *Faecalibacterium prausnitzii*, the sole identified species within the genus *Faecalibacterium*, is a well-characterized butyrate-producing bacterium with documented anti-inflammatory activity [78]. SCFAs may influence central nervous system function through multiple gut–brain communication pathways, including circulating immune and metabolic signals, the enteric nervous system, and vagal afferents. Clinical studies have further linked lower circulating SCFA concentrations to greater depressive symptom severity, whereas higher pretreatment concentrations have been associated with a more favorable therapeutic response [79]. Reduced abundance of *Faecalibacterium* has been linked to diminished anti-inflammatory capacity, impaired intestinal barrier integrity, and decreased regulatory T-cell differentiation [80,81,82], thereby potentially promoting systemic inflammation. Consistent with this, decreased *Faecalibacterium* abundance has been reported in patients with depression, whereas administration of *F. prausnitzii* has been reported to attenuate anxiety- and depression-like behaviors in animal models [57,81,83]. Moreover, previous evidence suggests that the relative abundance of *Faecalibacterium* is inversely related to depressive symptom severity [81]. Mechanistically, *Faecalibacterium* enhances intestinal barrier integrity through the production of SCFAs, particularly butyrate, thereby limiting lipopolysaccharide (LPS) translocation into the circulation. This, in turn, mitigate hypothalamic–pituitary–adrenal (HPA)-axis hyperactivity and the accompanying increase in cortisol secretion [84]. Other butyrate-producing taxa, including *Ruminococcaceae* and *Hungatella*, may exert similar protective effects [85,86,87]. In addition, *Akkermansia muciniphila*, a key regulator of the microbiota–gut–brain axis, has been implicated in maintaining intestinal barrier integrity, modulating host metabolism, and attenuating neuroinflammation [88]. Experimental evidence further indicates that *A. muciniphila* can promote neurogenesis and synaptic plasticity, with potential benefits for cognitive and emotional function [89].

Depletion of this species is not restricted to depression-related conditions but has also been reported in Alzheimer’s disease [90], Parkinson’s disease, and various neurodevelopmental disorders [91,92,93]. Consistent with prior reports, reduced levels of *Subdoligranulum* [94] and *Lachnoclostridium* [95] have been observed in MDD cohorts, while multi-cohort analyses have identified decreases in *Bacteroides_plebeius* and UCG_002 in depression-associated microbiota profiles [96]. Collectively, these findings suggest that depletion of SCFA-producing and barrier-supporting taxa may contribute to PPD pathophysiology through disruption of gut homeostasis, impaired barrier function, and enhanced inflammatory signaling along the gut–brain axis. Notably, *Erysipelatoclostridium* and *Prevotellaceae* were detected in both the HC and PPD groups, suggesting that their roles may be context-dependent rather than uniformly beneficial or detrimental. For instance, *Erysipelatoclostridium* has been reported to exert protective effects in Behcet’s disease (BD) [97], a systemic vasculitis. However, *Erysipelatoclostridium ramosum* has also been shown to promote intestinal serotonin (5-hydroxytryptamine) secretion by enterochromaffin cells [98]. Although peripheral serotonin does not readily cross the BBB, it may influence BBB permeability [99,100], potentially facilitating neuroinflammatory processes implicated in psychiatric disorders. Consistent with this dual role, increased *Erysipelatoclostridium* abundance has been reported in mild cognitive impairment and depression cohorts [101,102]. In contrast, *Prevotellaceae* has been associated with potential protective effects, with studies reporting a negative correlation between its abundance and PPD [103]. Its depletion has also been observed in MDD [104] and ASD [105]. Interestingly, *Prevotellaceae* abundance appears to be elevated in insomnia [106], further highlighting the context-specific and potentially heterogeneous roles of this taxon across neuropsychiatric conditions. From a nutritional perspective, the depletion of SCFA-producing and barrier-supporting taxa may be clinically relevant because these microbial groups may be modifiable through dietary fiber intake, prebiotic supplementation, fermented foods, and other microbiota-targeted nutritional strategies [35,107,108,109].

In addition, HAMD-17 scores were positively associated with several PPD-enriched taxa, including *Ralstonia*, *Fusobacterium*, and *Lactobacillus*, and negatively associated with HC-enriched taxa, including *Faecalibacterium*, *Akkermansia*, and *Hungatella*. These findings are consistent with previous reports linking reduced SCFA-producing bacteria, particularly *Faecalibacterium*, to greater depressive symptom severity [58,81]. However, these associations were exploratory and do not imply causality.

### 4.2. Metabolomic Analysis

Earlier research has indicated that serum metabolic profiling may aid in distinguishing individuals with MDD [110]. Accordingly, we performed differential analysis of serum metabolites and conducted correlation analyses to further elucidate the relationships between gut microbiota and metabolite profiles.

Given the untargeted nature of our LC–MS analysis, the identified differential metabolites should be interpreted as PPD-associated circulating metabolic features rather than exclusively as direct microbial metabolites. These features may reflect host metabolism, dietary exposure, xenobiotic-related signals, or host–microbial co-metabolic processes. Therefore, the microbiota–metabolite correlation analysis was used to identify co-varying microbial and metabolic features associated with PPD, rather than to determine the biological origin or microbial production of individual metabolites. Although alterations in the tryptophan–kynurenine pathway have been implicated in postpartum mood disorders, tryptophan-related metabolites did not meet the differential metabolite criteria in our analysis, possibly due to differences in analytical strategy, cohort characteristics, postpartum timing, sample size, statistical thresholds, and metabolite annotation coverage [111,112]. Therefore, future targeted metabolomics studies are warranted to evaluate this pathway more precisely in PPD.

*Lysophosphatidylethanolamine (LPE)* is a key intermediate in lipid metabolism with emerging relevance to neurobiological processes. Experimental evidence indicates that *LPE (16:0)* can promote neurite outgrowth via specific signaling pathways in cultured cortical neurons [113], suggesting a potential neuroprotective role. Consistent with this, our metabolomic analysis revealed higher levels of *LPE (0:0/16:0)* in the HC group compared with the PPD group. However, findings in the literature remain inconsistent. Elevated *LPE* levels have been reported in patients with MDD, where they positively correlate with symptom severity [114]. Moreover, alterations in *phosphatidylethanolamine (PE)* and *LPE* levels have been associated with disease progression in mild cognitive impairment, underscoring their broader relevance to neurodegenerative processes [115]. In the present study, the enrichment of *LPE (0:0/22:4)* in the HC group contrasts with previous reports, highlighting potential heterogeneity across cohorts. Lipid metabolic dysregulation has been increasingly implicated in depression, partly through its role in modulating peripheral and central immune-inflammatory responses that contribute to neuroinflammation [116]. Notably, gut microbiota are key regulators of host lipid metabolism. For example, *Fusobacterium nucleatum* has been shown to induce glycolysis and lipogenesis via activation of the PI3K/Akt/mTOR signaling pathway in hepatocytes [117], whereas *Akkermansia* supplementation ameliorates hepatic steatosis and inflammation in models of non-alcoholic fatty liver disease [118]. These findings suggest a potential association between gut microbiota-related lipid metabolic alterations and neuropsychiatric disorders. Consistent with this framework, correlation analyses revealed that taxa enriched in the PPD group, including *Blautia*, *Fusobacterium*, *Collinsella*, and *Ralstonia*, were positively associated with *LPE (0:0/22:4)* and *LPE (0:0/16:0)*. In contrast, taxa enriched in the HC group, such as *Faecalibacterium*, *Akkermansia*, *Hungatella*, *Prevotellamassilia_timonensis*, and *Ruminococcaceae*, exhibited negative correlations with these metabolites. These exploratory patterns suggest possible covariation between gut microbial composition and *LPE*-related serum metabolic features. However, they do not establish microbial regulation of lipid metabolism, and the direction, biological source, and consequences of these associations remain uncertain. These findings also suggest that lipid-related metabolic disturbances in PPD may be influenced not only by host neuroendocrine status but also by nutrition-sensitive microbial metabolism, including dietary fat quality and microbiota-mediated lipid transformation.

*Carboxyaminoimidazole riboside (AICAR)* is an endogenous activator of AMP-activated protein kinase (AMPK), a central regulator of cellular energy homeostasis and inflammation. Emerging evidence indicates that *AICAR* exerts anti-neuroinflammatory effects by modulating LRRK2 mRNA stability and reducing LRRK2 protein expression [119]. In addition, *AICAR* has demonstrated therapeutic potential in neurodegenerative models, including Alzheimer’s disease, where it attenuates microglia-mediated inflammation and oxidative stress [120]. Consistent with these findings, our data revealed elevated *AICAR* levels in the HC group, suggesting a potential protective role. Gut microbiota may contribute to this effect through modulation of *AICAR*-associated metabolic pathways. For instance, activation of AMPK signaling by Clostridium butyricum has been shown to alleviate disease progression in experimental models [121], while Ascophyllum nodosum polysaccharide (ANP) enhances *AICAR* biosynthesis via microbiota-dependent mechanisms and mitigates colonic inflammation [122]. These observations raise the possibility that *AICAR*-related metabolic alterations may be associated with inflammatory regulation in PPD; however, direct evidence in PPD remains lacking. Consistent with this framework, several differentially abundant taxa identified in our study have established anti-inflammatory properties. Supplementation with *Faecalibacterium prausnitzii* restores short-chain fatty acid levels, reduces depressive-like behaviors, and attenuates hippocampal inflammation in preclinical models [123]. Similarly, *Akkermansia muciniphila* has been shown to reduce systemic inflammation and improve metabolic parameters in humans [124], while *Hungatella* may exert protective effects through suppression of pro-inflammatory cytokines such as IL-18 [125]. In addition, the signal was putatively annotated as *4-chlorophenylacetic acid (4CPA)* and mapped to the tyrosine metabolism pathway. However, its chemical identity, biological origin, and relevance to depression-related processes remain uncertain and require targeted validation. Given that reduced circulating tyrosine levels have been associated with depression [126], alterations in *4CPA* may reflect disruptions in this pathway. However, our findings diverge from prior reports, indicating the need for further investigation. Notably, both *AICAR* and *4CPA* showed nominal associations with selected microbial taxa in the exploratory correlation analysis, as illustrated by the Sankey diagram. These observations support further investigation of possible microbiota–metabolite relationships but do not establish a functional link.

*Penitrem F* and *Testololactone* were also identified as differential serum metabolic features in the untargeted LC–MS-based analysis. Given their putative annotation and the absence of targeted confirmation, these features were interpreted cautiously and retained only as exploratory PPD-associated circulating metabolic features [127,128]. For *Testololactone*, we focused on the broader steroid-related metabolic context, given the relevance of postpartum endocrine changes to PPD [7,129]. The biological origins and clinical relevance of these features require further validation in future studies incorporating targeted metabolomics and detailed exposure assessment [127,128].

We further explored the nominal associations between HAMD-17 scores and key serum metabolic features within the PPD group. HAMD-17 scores were positively associated with several serum metabolic features, including *LPE* species, *Tyr-Thr-Gln-Arg*, *Penitrem F*, and *Testololactone*. Notably, previous lipidomic studies have reported associations between LPE/Lyso-PE species and depression severity, while metabolomic studies of PPD and MDD have implicated disturbances in lipid metabolism, amino acid metabolism, energy metabolism, and steroid-related pathways [47,114,130,131,132]. These findings suggest that serum metabolic alterations may be associated with depressive symptom severity in PPD, although they should be interpreted cautiously and require further validation.

### 4.3. ROC Analysis

A growing body of evidence has identified both microbial and metabolite signatures associated with depression [81,133,134,135,136,137]. In line with previous machine learning–based metagenomic studies identifying *Faecalibacterium prausnitzii* as a marker for depression [138], our findings also highlighted *Faecalibacterium* as a microbial feature with preliminary discriminative performance in PPD. Additionally, prior studies have reported that *Akkermansia* abundance correlates positively with depression severity, including HAMD-17 scores, in both major depressive disorder and post-stroke depression (PSD) cohorts [139,140]. Other taxa, including *Subdoligranulum*, *Hungatella*, and *Ruminococcaceae*, have similarly been associated with depressive symptoms [99]. Consistent with these observations, our results suggest that *Ralstonia*, *Fusobacteriota*, and *Pseudomonas* may represent candidate discriminative microbial features associated with PPD, although these findings require further validation. Serum metabolic features also showed preliminary discriminative performance in ROC analysis. LPEs have previously been proposed as early biomarkers for neurodegenerative and inflammatory conditions, including Alzheimer’s disease (AD) [77] and systemic lupus erythematosus (SLE) [141], which may support the exploratory relevance of lipid-related metabolic features in our cohort. In addition, steroid-related metabolic features may be biologically relevant to depression-related phenotypes, given the reported association between androgen fluctuations and depressive symptom [142,143,144]. Moreover, because candidate features were selected and evaluated within the same cohort, the ROC analyses may be susceptible to overfitting. However, given the relatively small sample size and the absence of external validation, cross-validation, or independent testing, the ROC results should be interpreted as exploratory rather than diagnostic. Therefore, these microbial taxa and serum metabolic features should be considered candidate discriminative features requiring further validation, rather than validated diagnostic biomarkers for PPD.

### 4.4. Limitations

Despite these promising findings, several limitations should be acknowledged. First, given the cross-sectional observational design, causal relationships among gut microbiota, circulating serum metabolic features, and PPD cannot be inferred. Therefore, the observed microbiome-metabolome associations should be interpreted as exploratory correlations rather than evidence of direct mechanistic or causal links. Second, the relatively small sample size, single geographic region, and species-level resolution of the gut microbiota may limit statistical power and generalizability. In addition, the use of 97% similarity-based OTU clustering provides lower taxonomic resolution and reproducibility than current ASV-based pipelines, such as DADA2 or Deblur. Therefore, the microbiome findings should be interpreted primarily at the community and genus levels, while species- or strain-level assignments should be considered tentative. Moreover, multiple-testing correction was not applied to the exploratory serum metabolic feature screening or correlation analyses, which may increase the risk of false-positive findings. Therefore, these results should be regarded as hypothesis-generating and require validation in larger independent cohorts using predefined multiple-testing procedures and targeted analyses. Third, patients with severe depressive symptoms were not included, as the eligible HAMD-17 range was 7–24; therefore, the findings may primarily apply to mild-to-moderate PPD. Fourth, although untargeted LC–MS-based serum metabolomics provides broad metabolic coverage, it has inherent limitations in metabolite annotation. Accordingly, putatively annotated drug- or xenobiotic-related serum metabolic features should be interpreted with caution. Their identities, origins, and biological relevance require further confirmation using targeted metabolomics and detailed exposure assessment. Finally, the internal stability of the ROC results was assessed using bootstrap resampling, and bootstrap-derived 95% confidence intervals were calculated for the AUC estimates. However, bootstrap assessment within the same cohort cannot replace external validation. Therefore, the candidate features remain exploratory rather than validated diagnostic biomarkers. Future longitudinal studies with larger independent cohorts, detailed postpartum phenotyping, targeted metabolomics validation, and mechanistic experiments are warranted.

## 5. Conclusions

β-diversity analysis revealed a distinct gut microbial community composition in participants with PPD compared with HC. Specifically, the relative abundances of *Faecalibacterium*, *Akkermansia*, and *Hungatella* were significantly reduced in the PPD group. Metabolomic analysis revealed that levels of *4-chlorophenylacetic acid, LPE (0:0/22:4)*, and *Penitrem F* were elevated in PPD, whereas *AICAR*, *Demethyldecarbamoylnovobiocin*, *LPE (0:0/16:0)*, *Progoitrin*, and *Testololactone* were decreased. Collectively, these findings suggest that PPD is associated with alterations in gut microbiota and metabolite profiles, including lipid-related, neuroinflammation, and steroid-related metabolic features. Several microbial taxa and putatively annotated serum metabolic features demonstrated exploratory discriminative potential for PPD. However, these candidate features require targeted validation in larger independent cohorts before their diagnostic or mechanistic relevance can be established. These findings may also provide preliminary clues for future nutrition-oriented strategies aimed at restoring microbial homeostasis and metabolic balance in women at risk of PPD, although prospective dietary and microbiota-targeted interventional studies are required.

## Figures and Tables

**Table 1:** Baseline characteristics and clinical symptoms of patients with PPD and HC.

Parameter	HC Group (*n* = 21)	PPD Group (*n* = 42)	*p*-Value
Sociodemographic profile			
Age, mean (SD), years	33.238 ± 3.590	34.191 ± 4.145	0.373
BMI, mean (SD),	21.640 ± 2.447	22.337 ± 3.136	0.376
High school or less, No. (%)	1 (4.8%)	4 (9.5%)	0.657
Menstruating, No. (%)	12 (57.1%)	25 (59.5%)	1.000
Constipation, No. (%)	5 (23.8%)	16 (38.1%)	0.396
Postpartum duration, mean (SD), day	142.857 ± 88.217	200.190 ± 125.300	0.329
Multiparity (≥2 deliveries), No. (%)	6 (28.6%)	20 (47.6%)	0.182
Cesarean delivery, No. (%)	7 (33.3%)	22 (52.4%)	0.187
Non-breastfeeding, No. (%)	15 (71.4%)	23 (54.8%)	0.277
Irregular dietary pattern, No. (%)	3 (14.3%)	8 (19.0%)	0.738
Severity of depressive symptoms			
HAMD-17	4.238 ± 1.670	13.381 ± 3.668	<0.01 *

HC, healthy controls; PPD, postpartum depression; HAMD-17, 17-item Hamilton Depression Rating Scale; * *p* < 0.05.

## Data Availability

The data presented in this study are available on request from the corresponding author due to privacy and ethical restrictions related to participant confidentiality.

## Supplementary Materials

The following supporting information can be downloaded at: https://www.mdpi.com/article/10.3390/nu18152562/s1, Table S1: Bootstrap-derived AUC estimates and 95% confidence intervals.

## Abbreviations

PPD	Postpartum depression
HC	Healthy controls
HAMD-17	17-item Hamilton Depression Rating Scale
ROC	Receiver operating characteristic
LPE	Lysophosphatidylethanolamine
AICAR	Carboxyaminoimidazole ribotide
CNS	Central nervous system
GABA	Gamma-aminobutyric acid
GAD	Gene encoding glutamate decarboxylase
SCFA	Short-chain fatty acids
BBB	Blood–brain barrier
DSM-5	Diagnostic and Statistical Manual of Mental Disorders
MDD	Major depressive disorder
OTU	Operational Taxonomic Units
PCoA	Principal coordinate analysis
LEfSe	Linear discriminant analysis effect size
LDA	Linear discriminant analysis
AUC	Under the curve
BMI	Body mass index
ASD	Autism spectrum disorder
LPS	Lipopolysaccharide
HPA	Hypothalamic–pituitary–adrenal
BD	Behçet’s disease
PE	Phosphatidylethanolamine
AMPK	AMP-activated protein kinase
ANP	Ascophyllum nodosum polysaccharide
4CPA	4-chlorophenylacetic acid
PSD	Post-stroke depression
AD	Alzheimer’s disease
SLE	Systemic lupus erythematosus
